# Oppositions, joints, and targets: the attractors that are the glue of social interactions

**DOI:** 10.3389/fnbeh.2024.1451283

**Published:** 2024-08-27

**Authors:** Jackson R. Ham, Sergio M. Pellis, Vivien C. Pellis

**Affiliations:** Department of Neuroscience, University of Lethbridge, Lethbridge, AB, Canada

**Keywords:** Eshkol–Wachmann movement notation, DeepLabCut, Australian magpies, Cape Barren geese, beluga whales, giant Madagascar hissing cockroaches, invariance, homeostasis

## Abstract

Social interactions are often analyzed by scoring segments of predefined behavior and then statistically assessing numerical and sequential patterns to identify the structure of the encounters. However, this approach can miss the dynamics of the animals’ relationship over the course of the encounter, one that often involves invariant bonds, say a nose-to-nose orientation, with many different movements performed by both partners acting to counteract each other’s attempts to break or maintain the relationship. Moreover, these invariant bonds can switch from one configuration to another during an interaction, leading from one stable configuration to another. It is this stepwise sequence of configurational stabilities that lead to functional outcomes, such as mating, aggression, or predation. By focusing on the sequence of invariant relational configurations, the deep structure of interactions can be discerned. This deep structure can then be used to differentiate between compensatory movements, no matter how seemingly stereotyped they may appear, from movement patterns which are restricted to a particular form when more than one option is available. A dynamic perspective requires suitable tools for analysis, and such tools are highlighted as needed in describing particular interactions.

## Introduction

Dyadic interactions between animals involve a complex web of interconnected movements by both partners. To capture the dynamics of such interactions, a common approach is for researchers to score predefined ‘behavior patterns’ and numerically evaluate the contribution of these by each animal (Pellis and Pellis, 2021). The simplest approach is to compare the frequency of performance of these behavior patterns over the course of the interaction by either one or both partners (e.g., Colvin, 1973; Dempster and Perrin, 1989; Fernández-Espejo and Mir, 1990). Deeper insight into the relative influence of the partners on each other can be gained by evaluating the temporal or sequential organization of the behavior patterns over the course of the interaction (e.g., Casarrubea et al., 2018; Clark and Moore, 1994; Donaldson et al., 2018; Lerwill and Makings, 1971). While we are not averse to using such methods (e.g., Cenni et al., 2020; Ham et al., 2023b; Hamilton et al., 2014; Lilley et al., 2020; Pellis and Pellis, 1983, 1992), our concern is that such methods can mask the true organizational structure of interactions.

**Figure 1:**
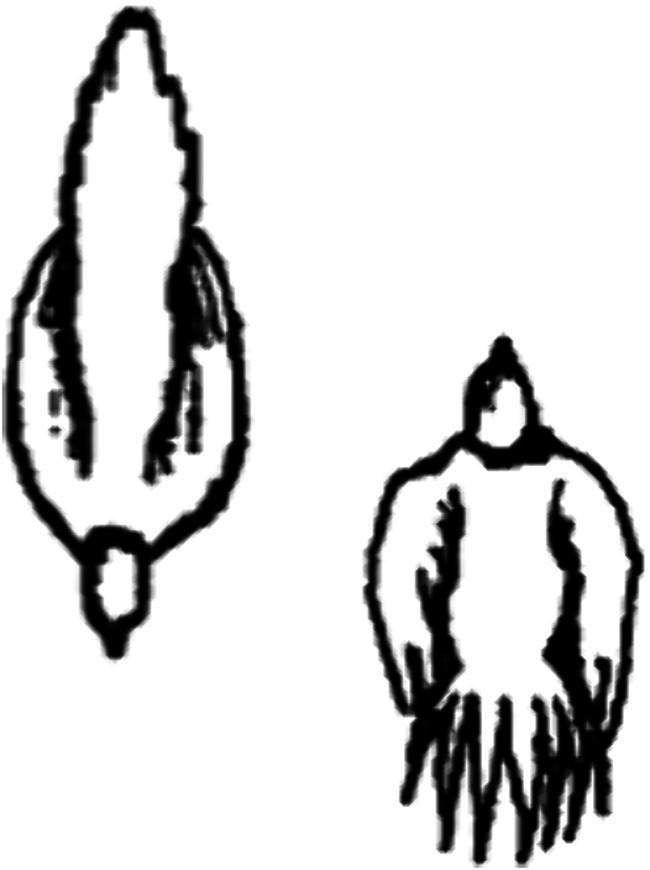
Drawn from an aerial perspective, the figure shows two greater sage grouse males in the so-called ‘facing past display’ (Reprinted from top panel of Figure 3, page 1581 from Pellis et al. (2013) with permission by Copyright Clearance Center).

The movements by the partners in an interaction may be continuous and overlapping, so carving up the encounter into discrete segments (i.e., behavior patterns variously defined) may be arbitrary, chosen for the ease of scoring by the researcher(s) rather than because they are biologically meaningful to the animals (Pellis and Pellis, 2021). More dynamic ways of tracking the movements by the partners may be needed to identify what is relevant to the animals themselves (Pfaus et al., 2023; Potegal and Nordman, 2023). For example, during the breeding season in greater sage grouse (*Centrocercus urophasianus*), a species from the plains of the interior west of North America (Schroeder et al., 1999), the males congregate in a lek, usually a slight rise or hillock on the prairie, where they perform courtship displays. Females inspect the lek, evaluate the males and pick their preferred male for copulation (Wiley, 1973a). As not all locations in the lek are equally propitious, males compete for occupancy of prime real estate, and do so by displaying and if necessary, fighting one another. Combat involves striking the opponent on the head with a wing, but often, simply adopting the ‘facing past display’ (FPD) suffices to induce the opponent to retreat (Wiley, 1973b). In the FPD, the two birds stand next to one another facing in opposite directions (anti-parallel) (Figure 1). Such a configuration may be considered as a display that affords the opponents the opportunity to size each other up and so decide whether it is worth escalating to combat, as has been argued for other species in similar situations (e.g., Clutton-Brock et al., 1979; Jennings et al., 2003). The frequency and duration of the FPD may be scored, as well as the temporal and sequential association of the FPD with other actions (e.g., strutting display, combat) performed by one or both interactants, rendering a plethora of numerical data with which to explore the structure of the encounters. But such an approach potentially misses the point.

The FPD is a by-product of the actions and counteractions by the interactants (Pellis et al., 2013). The optimal combat strike is for the attacker to hit its opponent on top of the head with a bony element of its wing, preferably the elbow, and the best position from which to launch such a strike is for it to be within no more than half a bird distance away, have its shoulder oppose its opponent’s head and be oriented slightly oblique to anti-parallel, bringing the wing a little closer toward the opponent’s head. But, of course, the opponent is not an inanimate puppet, but an agent, who will not only do what it can to prevent the other bird from gaining the best position from which to strike, but also will maneuver to gain the best position itself. This means that FPD is not a static posture by either animal, but a dynamically maintained one, arising from the moves and countermoves by both interactants. Once in an FPD, as one bird begins to turn away to leave, it places itself in a vulnerable position to be struck by the other bird, resulting in a stalemate, and so stuck in the FPD configuration. The stalemate is gradually broken after a prolonged period, with one bird making imperceptible, incremental movements away until it can turn and leave safely (Pellis et al., 2013). Several important conceptual and methodological lessons are illustrated by this example.

Abstracted behavior patterns may not capture the dynamics of the moves and countermoves by the two opponents, what may do so is the configurational relationship between the two animals. Conceptually, this means that the actions performed by each animal are in the service of gaining or maintaining some perceptual configuration with the other, which, in the case of the sage grouse, involves vacillating between maintaining the anti-parallel position to deny its opponent the advantage while simultaneously attempting to achieve the slightly off anti-parallel configuration from which to deliver a wing strike (Pellis et al., 2013). Consequently, actions by the animals need to be evaluated for their role as compensatory maneuvers to overcome the disruption to the preferred configuration due to the movements of the opponent—that is, the preferred perceptions are maintained by homeostasis (Powers, 2005). Methodologically, this raises the challenge to use measurement techniques that can track the relationships between the opponents’ bodies, how they change over the course of the encounter and determine whether specific actions by one animal are compensatory or independent of the other animal’s actions (Bell, 2014; Pellis and Bell, 2020; Pellis and Pellis, 2021). Golani (1976) introduced a framework that provided a practical way to tackle these important, but difficult conceptual and methodological issues in the study of animal behavior. To understand Golani’s conceptual innovations and how they impinge on studying interactions, we must first briefly examine the method he employed.

## Identifying the glue that binds interactions

The Eshkol–Wachmann Movement Notation (EWMN) (Eshkol and Wachmann, 1958) is a globographic system, designed to express relations and changes of relation between parts of the body, with the body treated as a system of articulated axes (i.e., body and limb segments). A limb is any part of a body that either lies between two joints or has a joint and an extremity. These are imagined as straight lines (axes) of constant length, which move with one end fixed to the center of a sphere. The body is represented on a horizontally ruled page into columns that denote units of time (e.g., frames of a video). The signs for movement are read from left to right and from bottom to top. Movements by any limb segment, or the body, can be described as the distal end moves across the surface of the sphere, with the proximal end being anchored in the center of the sphere. Typically, the locations on the sphere (horizontal and vertical) are at 45° angles, but the unit of angular measurement can be reduced (e.g., 22.5°) if finer grain comparisons are needed. An important feature of EWMN is that the same movements can be notated from several different perspectives: the coordinates for the position of the body segments can be scored with reference to the environment, to the body segment to which it is connected, and the movement by one animal can be described relative to the body of the other animal. By transforming the description of the same behavior from one coordinate system to the next, invariance in the behavior may emerge in some coordinates but not others (Golani, 1976). Such invariance may provide a clue as to the existence of the perceptions that one or both animals in an interaction maintain constant (Powers, 2005).

**Figure 2:**
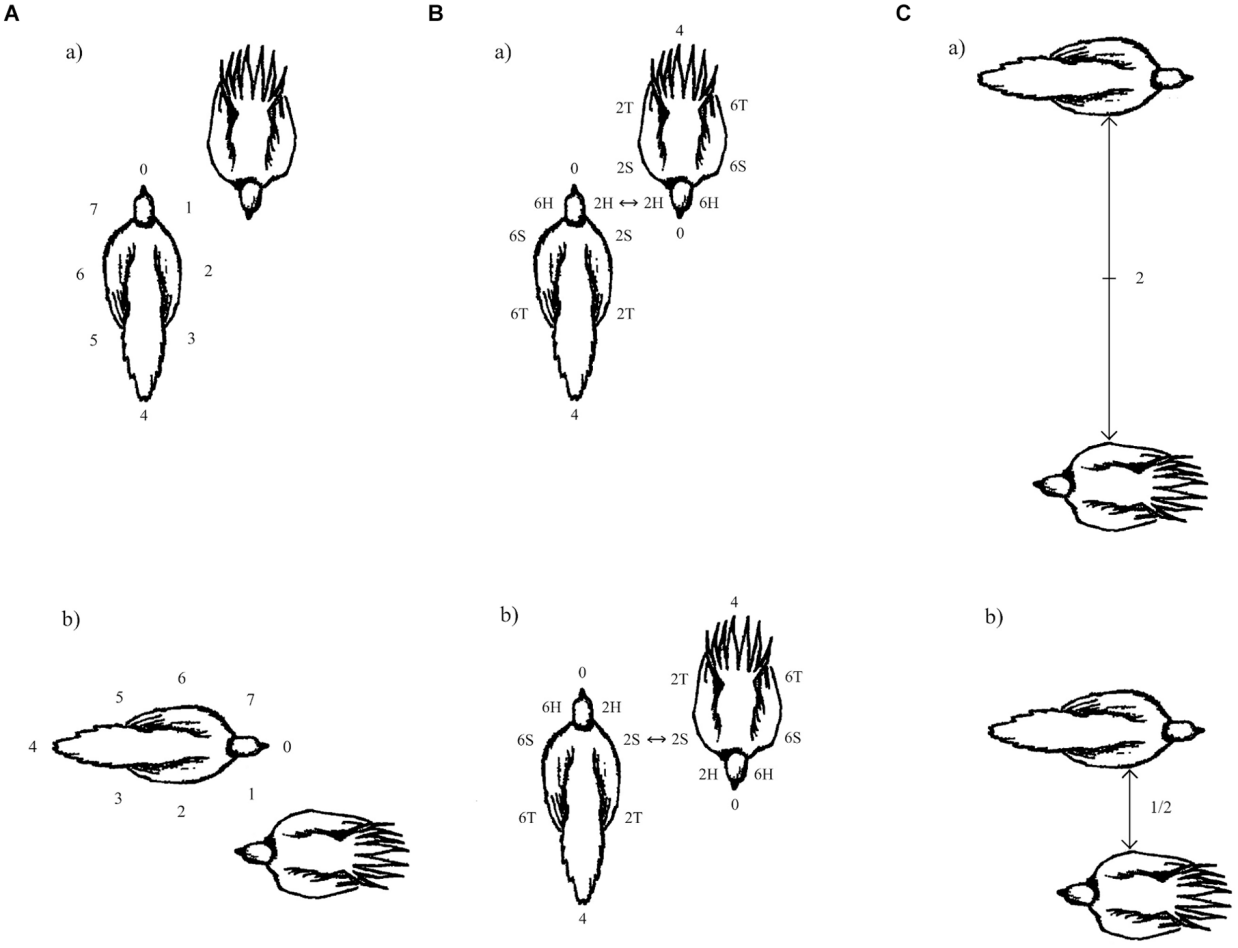
The figure illustrates the three, simplified, horizontal coordinates derived from EWMN that were used to record inter-animal configuration during interactions by male greater sage grouse. **(A)** Partner-wise orientation: In this case, even though the birds change their position in space, the relative orientation of their longitudinal axes remains the same. **(B)** Opposition: In this case, as the animals move, they switch from an opposition that is head-to-head to one that is shoulder-to-shoulder opposition. **(C)** Relative distance: In this case, as the birds move, they decrease their relative distance to one another. (Reprinted Figure A.1, page 1594 from Pellis et al. (2013) with permission by Copyright Clearance Center).

In interactions between two animals, three measures have proven to be particularly useful to track inter-animal relationships (e.g., Moran et al., 1981; Pellis, 1982). For simplicity, these measures are shown in an example in which the animals’ movements were only tracked in the horizontal plane as they were used in describing the FPD and combat in sage grouse (Pellis et al., 2013). The three measurements are:

1. Partnerwise orientation: This refers to the relationship of the longitudinal axis of one animal relative to the other. One animal is selected as the focal animal and the 45° units are situated in a circle around the longitudinal axis (0–7), with 0 being situated in the direction in which the animal is facing. Wherever the other animal is in space, its longitudinal axis is envisaged as transecting that of the focal animal carrying the EWMN coordinates, with the number pointed at by the anterior of the opponent being given that numerical value for the partnerwise orientation. For example, in Figure 2Aa, the focal animal (with the numerals surrounding its body) is facing upward on the page and the other animal is standing facing the bottom of the page, thus pointing in the direction of 4 on the focal animal, giving the pair a partnerwise angle of 4. Then, as the focal animal changes its position in space, so does the other animal, leading them to maintain the same partnerwise angle (Figure 2Ab).
2. Opposition: With this measure, the part of the body of one animal closest to the body part on another is scored. To score this, imagine the EWMN sphere being deflated, so that it is wrapped around each animal’s body. The front of the sphere (taking the horizontal value only) would be 0 and this value would be attached to the tip of the beak or snout, with the rearmost point as 4. Similarly, each side of the body (head, shoulder, torso) would be labeled 2 for the right side and 6 for the left side. The body parts opposed by the two animals can then be tracked during the encounter. For example, in Figure 2Ba, the two animals are standing in such a way so that the right sides of their heads are opposing one another (2H/2H). Then, as the animals move, the points on their bodies of closest opposition changes (Figure 2Bb) to the right side of their shoulders (2S/2S).
3. Relative distance: Given that videotapes are often not taken with a measurable frame of reference, the absolute distance in a metric, such as centimeters, is not possible, but the distance in terms of animal lengths (i.e., from the tip of snout or beak to the base of the tail when the animal is standing in a relaxed posture) can be used to track the relative distance, during encounters, between the animals. For example, in Figure 2Ca, the two animals are standing side-by-side, facing opposite directions and are two animal lengths apart. Then, following some movement by one or both animals, they maintain the same orientation, but move closer together (Figure 2Cb), ending up only half an animal distance apart.

**Figure 3:**
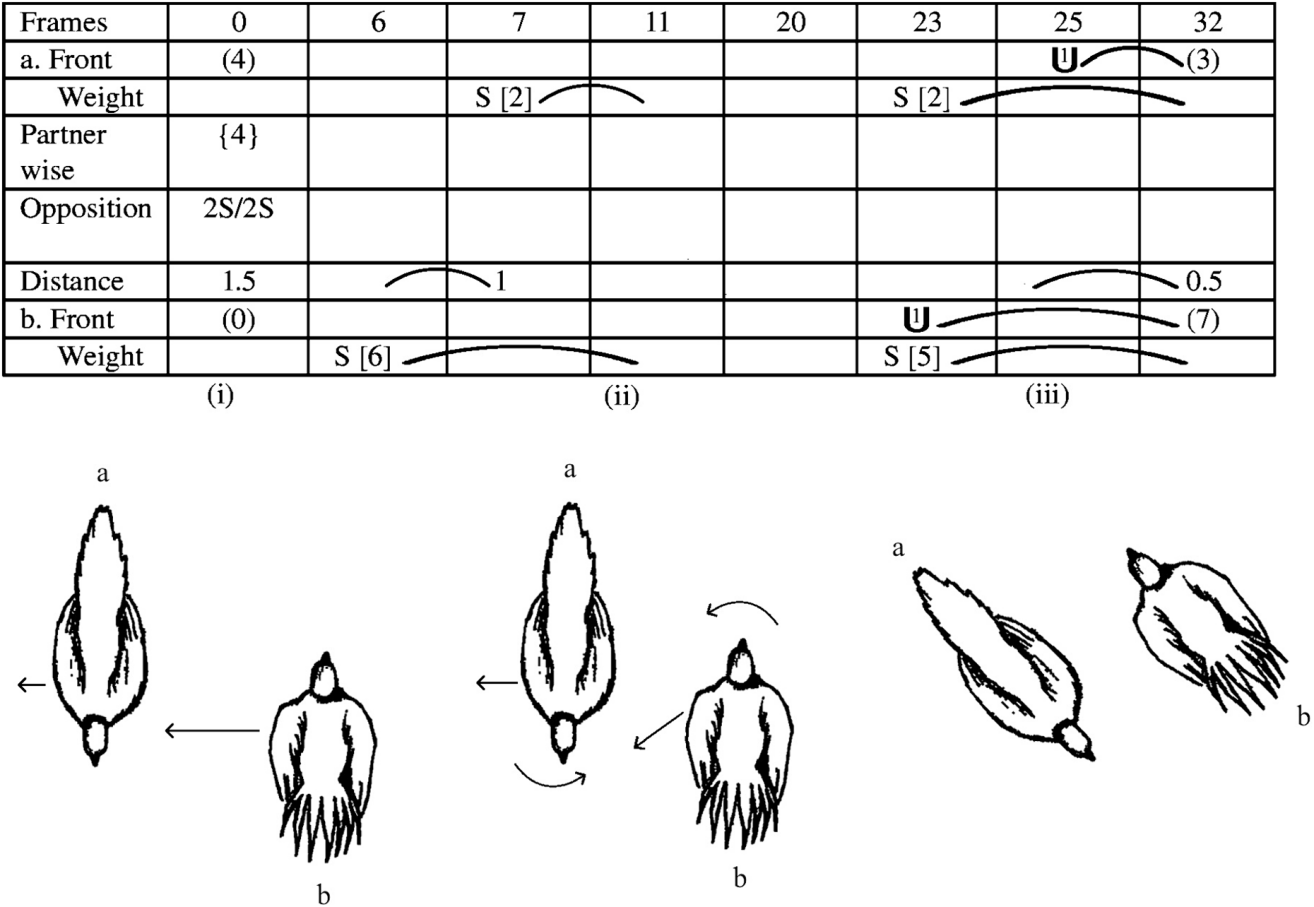
A short sequence, around one second, embedded within a fight, in which two clusters of movements by the two opponents are illustrated. (Top panel) The notated score for the movements. (Bottom panel) Drawings from a dorsal view of the relative positions of the birds at specific moments in the score. (Reprinted Figure A.2, page 1597 from Pellis et al. (2013) with permission by Copyright Clearance Center).

Combining these measurements enables us to track the inter-animal relationships between the birds as the interaction proceeds. However, something else is needed as well; the type, direction and magnitude of each bird’s movements in space, so that we can determine if a change occurs in one or more of the inter-animal measures, which member of the pair produced that change. Conversely, if, despite movements in space by both birds, the inter-animal measures remain unchanged, we are alerted to those movements being compensatory—movements by one bird are negated by movements of the other. These combined measurements are illustrated on a notated page for an FPD interaction (Figure 3 top panel). Note that the individual spatial movements by each bird are shown at the top and bottom of the page (for bird a and b, respectively), and that for simplicity, each bird’s spatial movements are captured by ‘Front’, which measures where in the surrounding space the bird is facing and ‘Weight’, which measures the direction of movement in space. In between, the three inter-animal measures are tracked. More detailed measurements of the bodily movements of each animal in an encounter can be tracked as needed by the researchers’ questions (e.g., Moran et al., 1981; Pellis, 1982), but to illustrate the basics of the methodology, we will simplify the actions by what can be captured by Weight and Front.

What can be discerned is that movements to close the distance or to alter the orientation by one bird are countered by movements of the other bird, resulting in the maintenance of the anti-parallel configuration, without either bird gaining undue advantage, which readers unfamiliar with EWMN can discern from the drawings of the birds in the bottom panel of Figure 3, with the arrows indicating the magnitude and direction of the movements performed by each bird. In panel (i), bird b moves laterally toward bird a, which then moves laterally away. However, the movement by bird b is greater in magnitude than that of bird a, leading to a reduced inter-animal distance at the end of their movements in panel (ii), which in the notated page is reflected by a 0.5 decrease in body length as bird b steps to its left (S [6]) in the Weight row, and bird a to its right (S [2]). Then, bird b rotates around its longitudinal axis, with its head moving toward bird a, and begins to step obliquely backwards toward bird a. In the notated page, these are shown by one unit of rotation (i.e., 45°) in the Front row and an oblique step to the left rear by bird b (S [5]) in the Weight row. However, as it does so, bird a also rotates around its longitudinal axis toward bird b and steps laterally away (S [2]), so that when the birds end their movements, as shown in panel (iii), even though they have changed their position in space, they have maintained the same relative inter-animal configuration.

There are many empirical papers illustrating the use of EWMN to identify the organization of behavioral sequences in both solitary and social behavior that readers can peruse (e.g., Eilam and Golani, 1989; Golani and Fentress, 1985; Golani et al., 1979; Moran et al., 1981; Pellis, 1981, 1982, 2011; Pellis and Pellis, 2016; Ottenheimer Carrier et al., 2015; Whishaw and Pellis, 1990; Yaniv and Golani, 1987). For readers interested in learning more about how to use EWMN, we recommend starting with Foroud and Pellis (2021) which provides a detailed description of the method as well as some training exercises. For the remainder of this paper, we focus on the conceptual derivatives from this approach to illustrate its uses and offer some extensions. It should also be noted that, in what follows, examples will be used from various species and behavioral contexts. The reason for this is four-fold. First, a major point we want to emphasize is that the methodological approach being proposed is not limited to any one species but can be used widely. Second, whether interactions are agonistic, as is the case for the sage grouse, or amicable, as in social play and sexual encounters (see below), interactions can be deconstructed into their constituent inter-animal invariants. Third, all the behaviors explored are naturally occurring ones that are biologically relevant to the animals, and in one way or another have been used to study the neurobiology and/or endocrinology of behavior, so should be of interest to a variety of behavioral neuroscientists. Fourth, there are limited studies involving the proposed methodology, so illustrating different aspects of the framework necessarily involves using the species/contexts most pertinent to the issue being considered. That is, there are major gaps in our knowledge for even intensively studied species and behaviors, such as in social play in rats (Achterberg and Vanderschuren, 2023; Pellis et al., 2022). For readers wishing to learn more about the biology of the species and behaviors discussed, we recommend that they consult the original empirical papers cited.

## The attractors of social interactions

**Figure 4:**
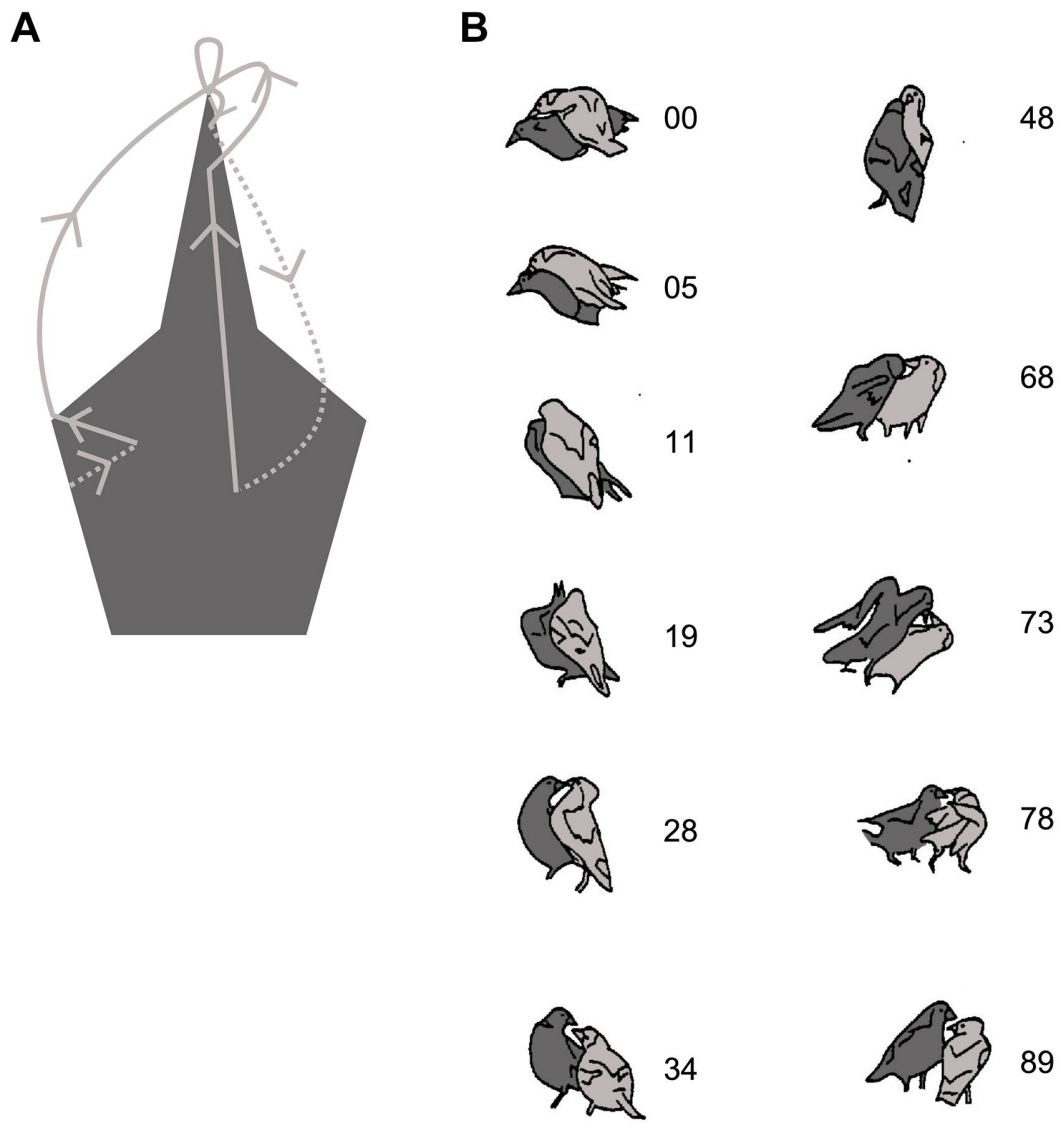
Play fighting in a pair of Australian magpies. **(A)** Shows a topographic summary of the oppositions between the tip of the attacker’s bill and the head of the defender. The oppositions oscillate between bill-to-side of head and bill-to-bill (see text). **(B)** The actual playful interaction is represented by drawings from frames of film (*ciné* film taken at 18 fps). The light gray bird starts with a bill-to-head opposition (with contact) with its partner (00) and maintains that opposition until the dark gray bird turns to face in a bill-to-bill opposition (28), which is maintained until it manages to gain a bill-to-head opposition (68). This is maintained until the light grey bird regains a bill-to-head opposition (89). (Adapted from Figure 4B, page 66 and from Figure 6, page 68 from Pellis (1981) with permission from Copyright Clearance Center). Created, in part, with BioRender.com.

A core inter-animal measure derived from EWMN is the opposition which traces how the bodies of the interactants oppose or contact one another over the course of the interaction (Figure 2A). One way to visualize the pattern of opposition and contact over the course of the interaction is to track the closest body part of one animal onto that of another. For example, play fighting in Australian magpies (*Gymnorhina tibicen*) involves the animals competing to peck each other on the head (Pellis, 1981). Tracking the tip of the bill of one bird relative to the head of the other bird shows that most of the changes in opposition arise from the movements of both birds (solid lines), with most ending in a bill-to-bill opposition, but when the bird represented does not move, the partner shifts the tip of its bill to the side of the other bird’s head (dashed lines) (Figure 4A). The diagram reveals two features of organization. First, the side of the head attracts the attacker and second, the bill-to-bill opposition is actively used by the defender to block the attacker from reaching the side of its head. The actual interaction, illustrated by whole body movements, shows how these oppositions were gained, maintained and changed (Figure 4B). The attacker (light gray) maneuvers to maintain its bill-to-head contact as the defender (dark gray) moves to break free.

Notating oppositions allows the observer to track the transitional connections between the two animals, and critically, to identify cases in which the animals become stuck at a particular bodily location, which acts like a virtual ‘joint’ between interactants (Golani, 1976). These body locations or joints that channel the animals’ actions can be thought of as ‘attractors’ (Golani, 1981), but of two distinct types. For the magpies, the one involving the bill-to-bill opposition is maintained jointly by both animals, like the case of the shoulder-to-shoulder opposition in the FPD of the sage grouse, whereas the bill-to-head opposition is maintained by one partner overcoming the maneuvers used by the other animal to break free from that opposition (Figure 4). By maintaining the bill-to-bill opposition, both animals are preventing the other from gaining access to the side of their head, whereas by moving to block the partner turning to face, the bird with the bill-to-head opposition is maintaining that opposition/contact. Thus, the bill-to-bill opposition is mutually beneficial, whereas the bill-to-head opposition is of benefit to the one that has the bill contact. So, joints may be a by-product of both animals competing to prevent one’s opponent from gaining the advantage while simultaneously maneuvering to gain the advantageous position, as illustrated by the FPD in the sage grouse (Pellis et al., 2013), or by one partner counteracting the other animal’s maneuvers to dislodge it from a favorable opposition (Pellis, 1981; Pellis et al., 2014). Therefore, notating oppositions is a useful way to detect joints (Golani, 1976, 1981), and joints provide clues as to the body targets that are attractors around which the interactions coalesce (Pellis and Bell, 2020).

**Figure 5:**
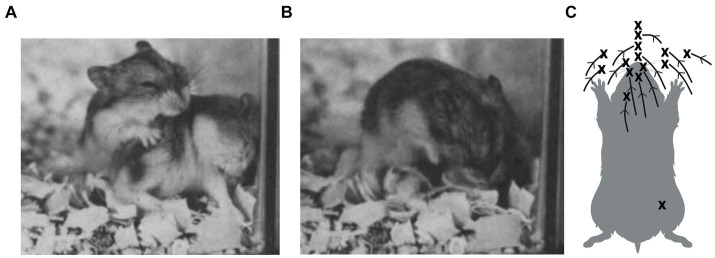
Play fighting in a pair of Djungarian hamsters shows one animal approaching from the rear and making mouth-to-shoulder contact **(A)**, then reaching over the top of its partner’s head towards its mouth, which the defender protects by tucking it into its chest **(B)**. In **(C)**, several attacks for a pair of male hamsters are summarized as changes in opposition on the dorsal surface of the animal being attacked. In virtually all cases, regardless of where on the dorsal surface contact begins, it is shifted forward towards the mouth, with X marking the end point. Failure to reach the mouth arises from the partner’s defensive actions, not from the attacker terminating the attack. (Panels A and B adapted from Figure 5, page 95 from Pellis (1988) with permission from John Wiley & Sons; and panel C adapted from Figure 2, page 234 from Pellis and Pellis (1989) with permission from John Wiley & Sons). Created, in part, with BioRender.com.

In competitive interactions, whether amicable, playful, predatory, or agonistic, members of a pair compete to gain and/or prevent access to a particular part of the opponent’s body (e.g., Aldis, 1975; Ben-David et al., 1991; Blanchard et al., 1977; Geist, 1965, 1967; Havkin and Fentress, 1985; Norman et al., 2015). Consequently, many of the actions performed during the interactions may be interpreted as tactics of attack and defense (Blanchard and Blanchard, 1994; Geist, 1966, 1978; Pellis, 1997; Pellis and Pellis, 2015). For example, during play fighting, Djungarian hamsters (*Phodopus campbelli*) compete to nuzzle and lick their partner’s mouth (Pellis and Pellis, 1989). One animal approaches and then reaches for the other’s mouth, while the other blocks the contact, and they then hold each other with their forepaws and reach for each other’s mouth. This continues until one succeeds in restraining and licking their partner’s mouth or fails to break through the other animal’s defense and walks off. Even when approaching from the rear, starting with contact lower on the dorsum of the recipient (Figure 5A), the attacker shifts that contact forward and over the top of the partner’s head toward the mouth (Figure 5B). A summary of multiple attacks from the rear tracking mouth-to-body opposition/contact, clearly shows how the partner’s mouth is the target that attracts the attack (Figure 5C). Identifying the targets allows observers to understand why certain behavior patterns occur in the contexts that they do. This is something that scoring behavior patterns independently of their context may fail to do or even be misleading. A couple of examples will illustrate the problem.

### Is it attack or is it defense?

For many species, both playful and serious combat involves biting, or otherwise striking, a particular body target on the opponent (Aldis, 1975; Blanchard and Blanchard, 1994; Blanchard et al., 1979; Cortés et al., 2024; Geist, 1966; Pellis, 1988, 1997). But biting takes two forms in agonistic interactions. For example, adult male rats may direct bites to the lower dorsum and flanks when an unfamiliar intruder is introduced into their home cage, and the intruder will direct retaliatory bites at the face of the attacking rat (Blanchard and Blanchard, 1990). Skins from free-living wild rats show the same pattern of bite-induced lesions (Blanchard et al., 1985). The pattern of wounding corresponds to the overt behavior exhibited during fighting—the attacker adopts tactics to gain access to their opponent’s lower dorsum and flanks and the defender adopts tactics to block access to those areas and may use retaliatory bites directed at the attacker’s face to do so (Blanchard et al., 1977; Pellis and Pellis, 1987). For species that compete to bite one another during play fighting, there is a similar division between offensive bites to the species-typical play targets and defensive bites directed at the attacker’s face (Kraus et al., 2019; Pellis and Pellis, 1997a; Pellis et al., 2014). A striking feature of play fighting is that partners not only compete for access to the species-typical bitten target, but they also incorporate some cooperation, resulting in play fights differing from serious fights in that they have some degree of reciprocity or turn taking (Palagi et al., 2016; Pellis and Pellis, 2017). However, there is variation across species in both how they incorporate cooperation and the degree to which they do so (Pellis et al., 2024).

One factor which influences the degree of cooperation is the rigidity of the social hierarchy (e.g., Ciani et al., 2012; Palagi and Cordoni, 2012; Petit et al., 2008; Reinhart et al., 2010). So, two measures need to be compared, the degree of symmetry in the play fights (i.e., the less symmetrical, the more competition relative to cooperation) and the steepness of the dominance hierarchy (i.e., the steeper, the more rigid the dominance relationships) (Cordoni and Palagi, 2016). We will focus on measuring play. One commonly used method is the play asymmetry index (PAI), which represents the proportion of offensive relative to defensive, and neutral behavior patterns occurring during play fighting and has been applied across a variety of species (e.g., Bagnato et al., 2023; Cordoni et al., 2016, 2018, 2021, 2022; Gallo et al., 2021; Llamazares-Martín et al., 2017; Nolfo et al., 2021). Biting, which is often categorized as an offensive behavior pattern in this index, is a problem, as has been noted above, biting can be delivered offensively or defensively. A further complication is that retaliatory bites may be delivered to the face, which is a defensive target, or at the species-typical play target, which would qualify as an offensive action. That is, retaliatory bites can be either offensive or defensive, with the relative proportion of each differing across species (Kraus et al., 2019; Reinhart et al., 2010). Consequently, lumping all bites into one category can distort species differences as measured by the PAI. The same applies to other actions occurring during social play which could be used for either attack or defense, such as grabbing and pushing. Given the focus of this paper on the role of oppositions, joints and targets in understanding the organization of social interactions, we will examine another behavior which occurs in playful combat which may be similarly ambiguous.

**Figure 6:**
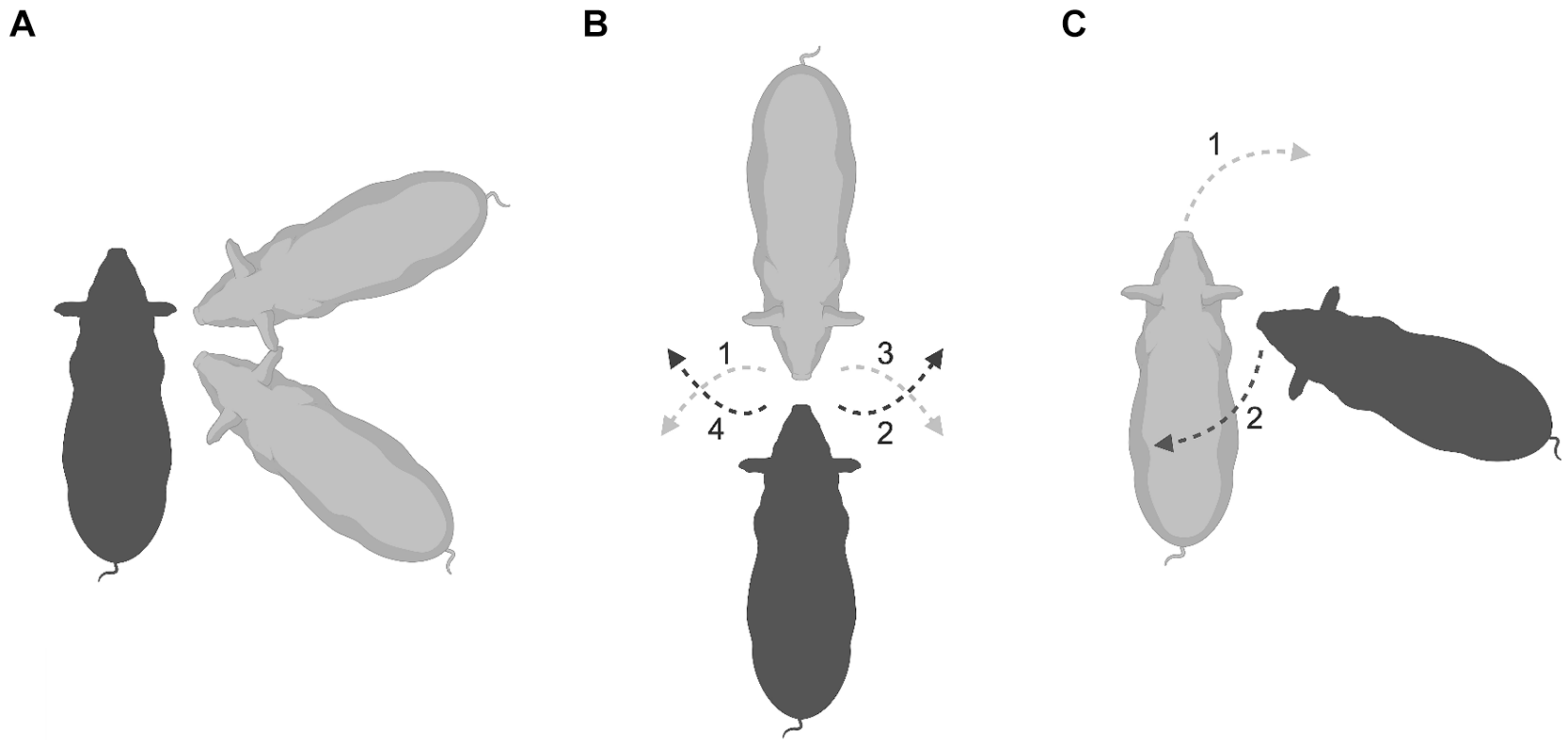
An aerial perspective is used to illustrate the positions of Visayan warty pigs during play fighting. **(A)** Shows the optimal position of the attacker (gray) when about to strike the defender (black), which can be either an oblique frontal or oblique rear orientation with its snout facing its partner’s shoulder. **(B)** Shows what happens when the defender (black) turns to face and so blocks its attacker (gray). From this mutual snout-to-snout opposition, moves by one animal to gain the optimal attack position are countered by the other (dashed lines), leading to the maintenance of a stable snout-to-snout joint. **(C)** Shows a defending pig (black) adopting what looks like an optimal attack configuration, pointing its snout toward its attacker’s shoulder (gray). However, it does not attack, but rather, maintains this position by countering its attacker’s movements to swing around and gain access to the defender’s shoulders (dashed lines). Created with BioRender.com.

Fighting in pigs (Suidae), both playful and serious, involves face-to-face wrestling with biting and slashing of the opponent’s sides of the face, neck and shoulders (Barrette, 1986; Cumming, 1984; Estes, 1993; Frädrich, 1974; Newberry et al., 1988; Rushen and Pajor, 1987; Šilerová et al., 2010). An analysis of play fighting in juvenile Visayan warty pigs (*Sus cebifrons*) using EWMN (Pellis and Pellis, 2016) revealed that when executing combat maneuvers, the optimal opposition from which to strike the opponent is from an oblique frontal or oblique rear angle with the snout opposing the partner’s shoulder (Figure 6A). Just as in magpies, to prevent one’s partner from gaining this optimal position, the other animal faces their opponent. Once in a snout-to-snout opposition (Figure 6B), if the attacker (gray pig) rotates to the right to face their opponent’s shoulder obliquely (gray dashed arrow 1), the defender (black pig) rotates to its right to maintain the snout-to-snout opposition (black dashed arrow 2). Similarly, if the gray pig rotates to its left (gray dashed arrow 3), the defender rotates to its left (black dashed arrow 4). Thus, an attempt by one pig to gain the advantage is blocked by a countermove by its opponent. Occasionally, something unexpected happens; one animal orients itself to oppose the other’s shoulder but does not attack. Closer analysis of these instances using EWMN showed that this opposition is maintained as a joint, with the one establishing that opposition countering the moves of the other animal to extricate itself (Figure 6C). Again, consider the gray pig as the attacker and the black pig as the defender. If the attacker makes a sudden rotation forward and around toward the defender’s right shoulder (gray dashed arrow 1), not only does the defender rotate to its left (black dashed arrow 2) to maintain its opposition toward the attacker’s shoulder, but it may also lunge forward toward the black pig’s shoulder, as a feint. This suggests that the black pig is using the threat of launching an attack at the target area as a means of defense. On casual inspection, it appears that the pig adopting the defensive opposition is often the smaller or less dominant member of the pair and is using this defensive ploy to gain respite from vigorous competitive fighting often associated with the snout-to-snout opposition (Figure 6B). What explains shifting to this snout-to-shoulder opposition as a defensive joint remains to be empirically determined, but it raises a methodological issue relevant to this present paper. Even though facing the opponent’s shoulder is most often an offensive behavior pattern, arbitrarily scoring it as such, as in the case of biting (see above), confounds its offensive and defensive uses (Figures 6A versus 6C), leading to measures such as the PAI being potentially misleading.

Defining behavior patterns as abstracted behavioral markers in an ethogram and then scoring them without taking the context in which they occur into account can lead to misinterpreting their causal functions within social interactions (Golani, 1976; Golani and Moran, 1983; Pellis and Pellis, 2021). Oppositions, joints and targets provide important contextual information for interpreting the actions that occur during social interactions (Moran et al., 1981; Pellis et al., 2013) and how changing motivations may transform the sequence of interaction.

### A motivational change in attack or in defense?

One source of motivational change is the hormonal one that occurs in many animals over the mating period. For instance, as the eggs maturing in female birds become progressively closer to the point of being primed for fertilization, females are increasingly receptive to copulation with a suitable male. These underlying hormonal changes are reflected in the unfolding of courtship behavior (e.g., Dong et al., 2013; Erpino, 1969; Fabricius and Jansson, 1963; Murton et al., 1969). Over the course of the breeding season, courtship interactions between pairs of bonded Cape Barren geese (*Cereopsis novaehollandiae*) increase in frequency and intensity, and most critically, the oppositions involved shift from one body location to another, until copulation and egg laying occurs (Pellis, 1982).

Both sexes can initiate contact by approaching and nibbling their partner’s tail feathers, and then the male, but not the female, may switch to nibbling the base of her neck or shoulder area. If the female squats, the male can stand on her back and shift its bill contact to the top of her head, from which configuration copulation can ensue. How the female responds to the male’s contact changes as the breeding season progresses. As this study was of free-living geese, hormonal data were not available, so the courtship interactions and copulations were back-dated from the date of egg-laying to compare different stages of the reproductive cycle. These were subdivided into (1) 3 months preceding nest-building, (2) the week preceding nest-building, (3) first week of nest-building, (4) second week of nest-building, (5) third week of nest-building, and (6) the week or weeks of egg-laying. Lining the nest with down and sitting on the nest began by the third week of nest-building (Pellis, 1982). In the first phase, most interactions were initiated by the female (78.6%), but by the second phase, most were initiated by the male (89.5%) and mostly by the male thereafter, and it was the female that actively defended herself from being contacted. The interplay between the moves and countermoves by the pair mates was notated using EWMN, capturing the inter-animal relationship and the movements by each partner (Pellis, 1982 and see Figures 2, 3 above for how this was tracked).

**Figure 7:**
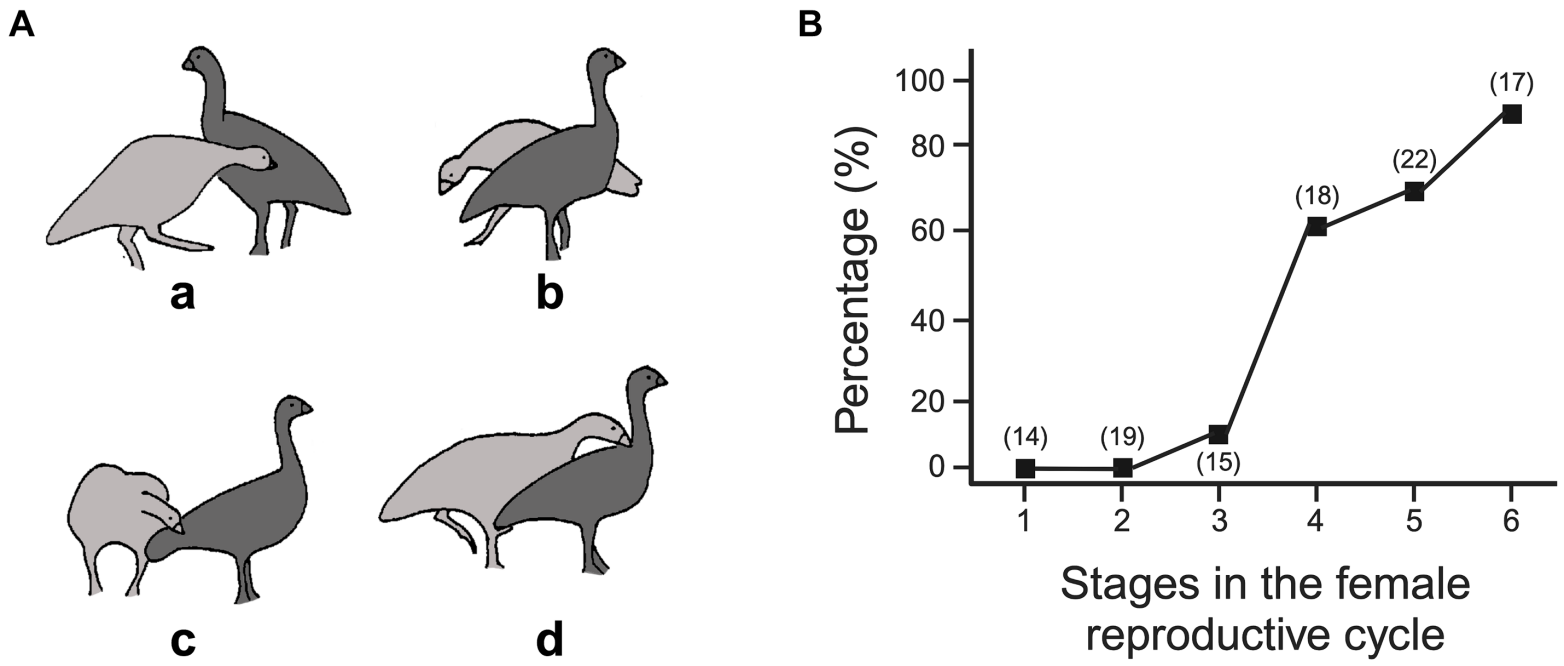
Courtship interactions in Cape Barren geese. **(A)** Shows the main phases of courtship, with drawings from 18 fps ciné film, starting with the male (light gray), with its head down, moving towards the female’s rump (dark gray) (see text for description). **(B)** Shows the probability that contact by the male leads to the female remaining stationary or squatting over the course of the mating season, with period 6 representing egg laying and nesting (see text). (Panel A adapted from Figure 3, page 33 and panel B derived from Table 3, page 39 from Pellis (1982) with permission from Copyright Clearance Center). Created, in part, with BioRender.com.

An interaction is illustrated in Figure 7A. The male goose (light gray) approaches the female (dark gray), lowers his neck, and orients his bill toward her rump (a), but as he gets close to making contact, the female rotates, pivoting her rump away from the male (b). The male keeps following the female and so circles around her to maintain the bill-to-rump opposition (c). Then, as the female slows down or stops, the male shifts bill contact, to the base of her neck (d). Depending on the female’s evasive maneuver, the male’s counter maneuver follows suite. If she walks away, he follows in a straight line, if she zig-zags, he zig-zags and if she rotates, as in Figure 7A, he circles. As she gets closer to egg-laying, the distance at which she begins to evade contact from the male decreases—so that, early in the mating season, the male following in a straight line after the female is most common, then later, as the distance at which evasion commences narrows, following the female’s zigzags are the most common, and then, finally, circling becomes the most common path. The decreasing distance at which the female begins to evade the male likely reflects her increasing sexual motivation, as is reflected in the increasing likelihood that contact by the male results in the female standing still or crouching (Figure 7B). Thus, as the breeding season progresses, the joints attracting the male progressively changes, with rump contact being initially sufficient, to rump contact leading to shoulder contact. Critically, the actions performed by the male in gaining and maintaining these joints are determined by the changing motivational state of the female. That is, the ‘attack’ behavior of the male is modified by the defensive behavior adopted by the female. Scoring the males’ behavior, such as following, zigzagging, circling, tail or shoulder pecking, independently of the context created by the females’ behavior would mask the causal processes involved (Pellis and Pellis, 2021). In this way, the virtual joints between the interactants are an essential measure to understand the behavior patterns performed by the individuals (Golani, 1976), as are the physical joints that lead to coordinated intra-body movements (Teitelbaum and Pellis, 1992; Whishaw et al., 1991). Another context in which oppositions can change over a protracted period and so shape the organization of interactions, is during development.

### A developmental change in attack or in defense?

Many behavior patterns, even complex social ones, do not need to be learned or practiced, to develop their species-typical form (e.g., Kruijt, 1964; Groothuis, 1993). For example, in rats, play fighting begins to emerge in the third week post birth and achieves its juvenile typical form by the end of the fourth week (Baenninger, 1967; Bolles and Woods, 1964; Pellis and Pellis, 1997b; Thiels et al., 1990). While how often play is performed as juveniles can vary based on experiences gained or missed in the first 3 weeks of postnatal life (e.g., Aguilar et al., 2009; Arnold and Siviy, 2002; Parent and Meaney, 2008; Shimozuru et al., 2007; Siviy and Harrison, 2008; Van Hasselt et al., 2012; Veenema and Neumann, 2008), the behavior patterns used during play fighting are only slightly modified if at all (Himmler et al., 2015; Himmler S. M. et al., 2014; Siviy et al., 2017). But changes with age in social interactions cannot be assumed to be independent of the actions of the social partner. For example, when rats find a small piece of food, they hold it in both forepaws, lean back onto their hind feet and eat the item. Rats, being social, will approach a partner that is eating a piece of food, reach over to sniff the item, and then attempt to rob them of it. The rat holding the food will evade this by pivoting around a vertical axis along the length of its body (“dodging”) and so laterally move its mouth away from the other rat (Whishaw, 1988). When rats dodge away from a male robber, defenders end their dodge so that their rump opposes the robber’s face, rather than the robber’s mid-flank area as is the case with female robbers (Field et al., 1997). If reared in social isolation over the juvenile period, when such dodging is quite common, rats can still execute the dodging behavior pattern as adults, except that its modulation relative to the robber is impaired (Pellis et al., 1999). Ending the dodge so that the rump faces the robber’s head (small target) is more difficult than facing the robber’s mid-body (large target), requiring greater coordination between the animals’ movements (Himmler B. T. et al., 2014). That is, the behavior pattern can be executed as normal, but its orientation is impoverished without the experience of dodging at younger ages.

**Figure 8:**
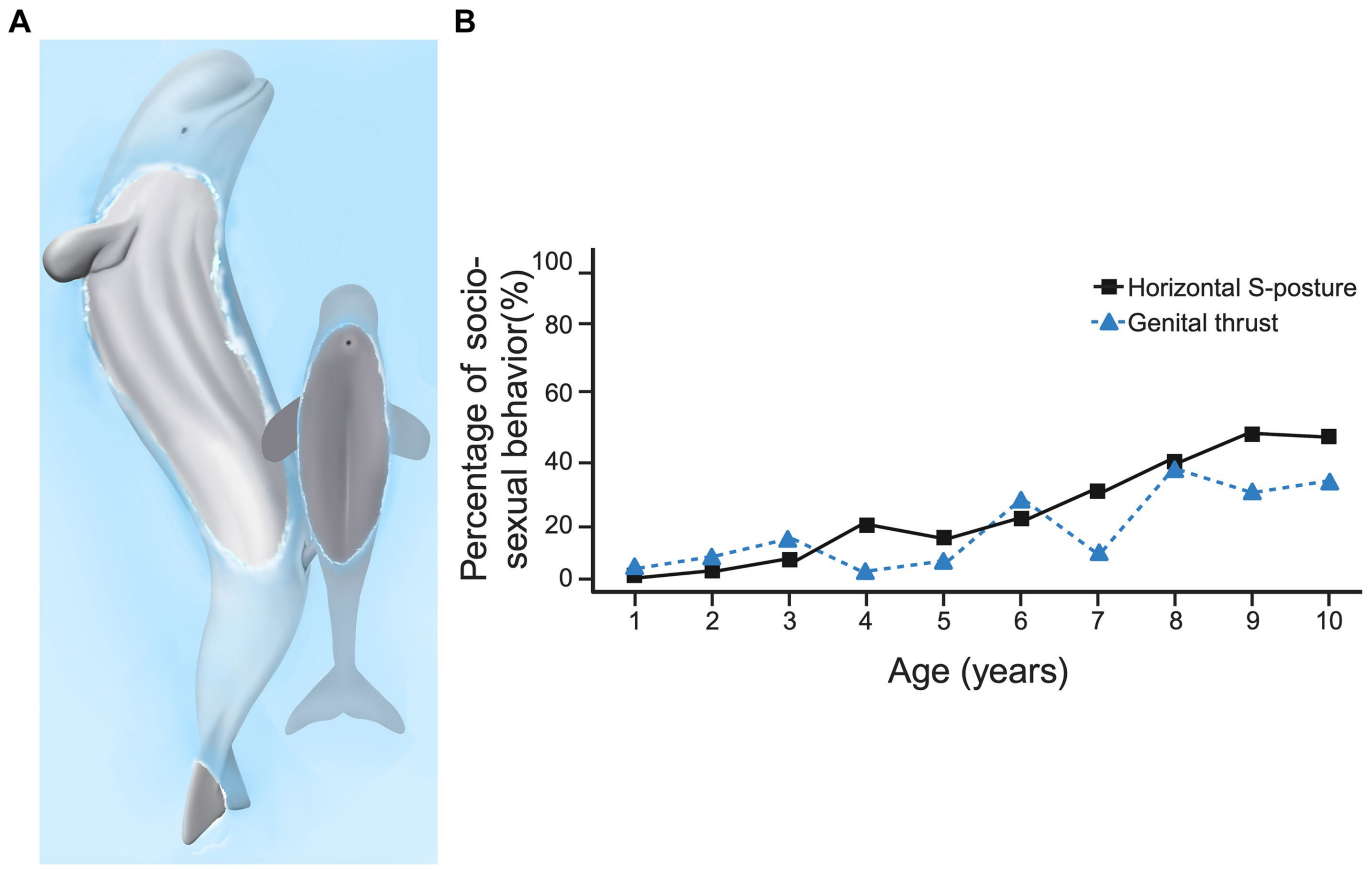
Socio-sexual behavior in beluga whales with **(A)** showing an adult male beluga thrusting, with a penile erection, towards a juvenile whale. The adult makes genital contact on the calf’s side. **(B)** Shows the percentage of male socio-sexual behavior that consist of horizontal S-postures and genital thrusts over the first 10 years. Panel **(A)** is adapted from Ham, Lilley and Manitzas Hill, (2023) (copyright held by original authors). Panel **(B)** combines quantitative data provided by Ham et al. (2022) and Lilley et al. (2020). For a detailed review of beluga socio-sexual behaviors and their development, see Manitzas Hill et al. (2024). Created, in part, with BioRender.com.

When an action by one animal needs to be coordinated with that of another, simply scoring the frequency of occurrence of that behavior over age may miss how that behavior is modified over development. For example, non-conceptive socio-sexual behavior is common across a variety of cetaceans (Ham et al., 2023a; Manitzas Hill et al., 2023a), and many socio-sexual behavior patterns are performed during play (Da Silva and Spinelli, 2023; Hill et al., 2015). In beluga whales (*Delphinapterus leucas*), playful socio-sexual behavior begins to be performed in the first year after birth, the various behavior patterns involved mature in their form until young adulthood (Ham et al., 2022; Hill et al., 2022; Lilley et al., 2020; Manitzas Hill et al., 2023b). Several behavior patterns are gradually coalesced into a functional unit. For example, in beluga whales, the S-posture, which involves bending the long axis dorso-ventrally into an S shape, can be performed as an independent display (Lilley et al., 2022) or can be integrated with genital rubbing and attempted intromission (Figure 8A). Not only does this require coordinating different body parts into an integrated action (the S-posture), but also coordination with the movements and position of the other animal to contact specific parts of their bodies (i.e., the genital area). Scoring the occurrence of the S-posture and the occurrence of genital rubbing in male belugas shows that these increase with age (Figure 8B).

**Figure 9:**
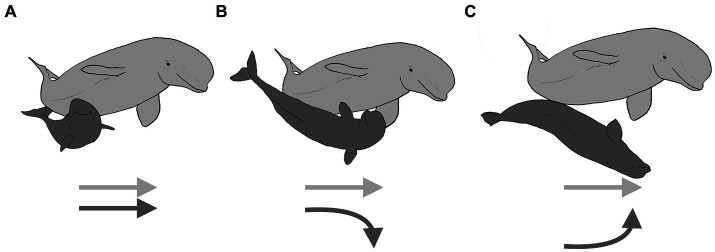
Playful socio-sexual behavior in beluga whales is shown with an older male (light gray) thrusting its genital area toward a calf (dark gray). Three alternative ways that the recipient can respond are illustrated: in panel **(A)**, the calf that does not respond to the playful thrust and instead continues swimming in the same direction it was before the thrust occurred, in panel **(B)**, the calf avoids genital-to-genital contact by rotating his ventrum away from the thruster, leading to genital-to-dorsum contact, and in panel **(C)**, the calf facilitates genital-to-genital contact by rotating to face the thruster. The arrows illustrate the relative movements by the interactants. Created with BioRender.com.

Despite the increasing frequency of performance of socio-sexual behaviors with age (Figure 8B), successful genital-to-genital contact is sporadic at all ages. Preliminary analysis of successful and unsuccessful contacts with EWMN, revealed that the recipient of a socio-sexual advance could act in one of three ways. The recipient of a sexual play thrust could do nothing, continuing to engage in the behavior they were doing before the playful thrust was launched (Figure 9A, with the dark gray recipient shown unchanging relative to the dark gray instigator by way of their corresponding arrows). Alternatively, the recipient could defend its genital area by rotating away from the thruster, juxtaposing its dorsal surface between its genitals and that of the attacker (Figure 9B, as indicated by the dark gray arrow moving away from the light gray arrow), or the recipient of the attack could rotate to face the thruster, facilitating genital-to-genital contact (Figure 9C, as indicated by the dark gray arrow moving toward the light gray arrow). Consequently, even though the S-posture seemingly matures with age (Ham et al., 2022), both this maturation and the continued low incidence of genital-to-genital contact between partners could arise from changes in the dynamics created by changes in the defensive responses of the recipients.

Because the recipient of a playful thrust can respond in multiple ways, a successful thrust, in which the two make genital-genital contact, is not completely explained by the competency of the thruster. That is, the partner can either facilitate or block the thruster from gaining an advantage by moving their genital area toward or away from the thruster regardless of the thruster’s proficiency. If only the successful genital-to-genital thrusts are scored, it remains unclear if the success is explained by improved motor coordination of the thruster (i.e., a developmental explanation), if the partner is modulating their behavior to challenge or facilitate their partner’s thrusts (i.e., an inter-animal cooperation explanation), or a combination of both. To understand both the developmental trajectory of the socio-sexual behavior patterns fully and the age-related changes in the interactions, both animals’ actions need to be tracked. A detailed EWMN analysis has yet to be completed, but the example illustrates that the oppositions created by the movements of both animals are needed to interpret the developmental changes in the behavior.

The examples used so far illustrate Golani’s (1976, 1981) main point, that for social interactions, what binds them together are the inter-animal relationships maintained and changed during the sequence. Scoring predefined behavior patterns may be helpful or they may mask the underlying structure of the interaction. To determine the difference, the inter-animal relationships need to be tracked to differentiate between the actions performed by one animal as a countermeasure to the movements of its opponent versus actions that are independent of those of the opponent. Hopefully, the examples provided above illustrate EWMN can be useful in this regard. However, there are at least two limitations to the widespread use of EWMN. First, it takes a long time to learn to use this method effectively, and analyzing filmed sequences is highly time consuming, limiting the number of sequences that can be analyzed. Second, like any useful tool, the pattern initially identified using EWMN may blind you from seeing alternative organizational features of the encounters. In the following section, we will use another example to illustrate how combining EWMN with novel AI approaches can mitigate these limitations.

## A way to the future: using AI to assess inter-animal coordination

During combat, male giant Madagascar hissing cockroaches (*Gromphadorhina portentosa*), slam each with their heads, and these have variously been labeled as ‘ram,’ ‘butt,’ and ‘lunge.’ In addition, the cockroaches can also contact one another with ‘bite’ and with the ends of their abdomens, with an ‘abdominal flick’ or ‘abdominal push’ (e.g., Barth, 1968; Breed et al., 1981; Clark and Moore, 1994; Nelson and Fraser, 1980). Irrespective of whether the head butts are directed at their opponent’s head or flanks, typically, butts are simply scored as butts. Given the examples described above, videotaped staged encounters between pairs of cockroaches were analyzed using EWMN to determine whether attackers randomly butt any accessible area of their opponent’s body (Bell, 2013, 2014). Tracking the inter-animal relationships and bodily movements by the opponents (as shown in Figures 2, 3) revealed that the animals maneuver to access their opponent’s flank. If the attacker successfully wedges the anterior of its head shield, which protrudes forward with a slight upward curve, beneath the lateral edge of its opponent’s body, thus between it and the ground, it can flip the defender over. Once flipped over, the attacker can bite the helpless cockroach on the exposed, softer undersides of its body. The defender can counter this maneuver by rotating around its longitudinal axis, pressing the lateral edge of its body facing the opponent against the ground. Quantitative scoring of butts to various locations on the flanks and the front of the head confirmed that contact with the lower flanks was significantly more frequent, and most critically, contact with the lower flanks was significantly more likely to lead to a flip over (Bell, 2014; Pellis and Bell, 2020). So, why target the head? They do not.

EWMN analyses of combat sequences showed that as one cockroach circles to gain access to the other’s flank, its opponent rotates to face the approaching animal and so block access to its flanks. This results in the two cockroaches facing one another. Once the heads are in opposition, they close contact and push one another, which, as in the Visayan pigs (Pellis and Pellis, 2016), if one’s postural stability is compromised, its partner uses the opportunity to circle to a flank. In this way, head-to-head butts are the by-product of the two animals countering one another, whereas head-to-flank butts are a product of one cockroach maintaining the optimal position for attack. Thus, EWMN revealed that interactions vacillated between head-to-head and head-to-flank oppositions, and these accounted for many of the actions performed by the interactants, as a maneuver by one was counteracted by the other (Pellis and Bell, 2020). Scoring butts, rams, or lunges independently of their dynamic context, as reflected by oppositions, confounds these differences by lumping them together into one numerical score. Again, EWMN has proved its worth, but can we improve on the limitations associated with EWMN?

### DeepLabCut and replication, replication, replication

A major advantage in using EWMN is that, unlike some computerized digitizing systems (e.g., Field et al., 1996), the metric space within which the behavior is filmed does not need to be known, making the system applicable to film collected in naturalistic settings (Golani, 1976). But, as noted above, a major limitation in using EWMN is the time it takes to notate sequences. Depending on the film rate used (e.g., 30, 60, 120 frames per second), means that for just 1 s of behavior, 30–120 frames need to be notated. For a behavioral sequence that takes many seconds or minutes to complete, notating can become a major chore, and detecting patterns of coordinated inter-animal movements on the resulting notated score sheets can tax the limits of even a seasoned notator, much less a relative novice (Pellis, 1981, 1982). A solution is to limit notation to a few exemplar sequences, identify the oppositions and joints that are maintained, and then test their robustness by scoring some static behaviors (i.e., behavior patterns that are correlated with the adoption of a specific opposition). If the pattern extracted from the notated sequences is real, then the quantitative scores should be as predicted (Pellis et al., 2013). Where possible, this follow-up testing can be done with a computerized digitizing system if the metric space within which the behavior occurs can be mapped, yielding interval scale measures rather than nominal or ordinal ones, increasing the precision in the way the behavior is assessed (Bell and Pellis, 2011).

Some modern AI based machine learning systems have resolved the problem of being limited to a known metrical space. That is, the computer program can learn to identify patterns, such as specific actions or bodily configurations, from regular video footage, which means that after a training period, the computer can track thousands of video frames and reliably detect patterns (e.g., Ardoin and Sueur, 2024; Chen et al., 2023; Eisdorfer et al., 2022; Inayat et al., 2020; Pereira et al., 2022; Wiltshire et al., 2023). Using such an approach means that an initial insight gained from EWMN analysis based on a few sequences can then be tested with a large random sample of sequences. As noted above, EWMN analysis showed that the Madagascar hissing cockroaches vacillated between head-to-head and head-to-flank oppositions, with limited manual quantitative scoring showing a bi-modal distribution—a small peak to the head and a large peak to the lower abdomen (Bell, 2013, 2014; Pellis and Bell, 2020). To test whether this pattern held up to a larger sample of interactions, we used multi-animal DeepLabCut (Lauer et al., 2022; Mathis et al., 2018) to score multiple encounters with many iterations of combat over many minutes resulting in thousands of video frames.

**Figure 10:**
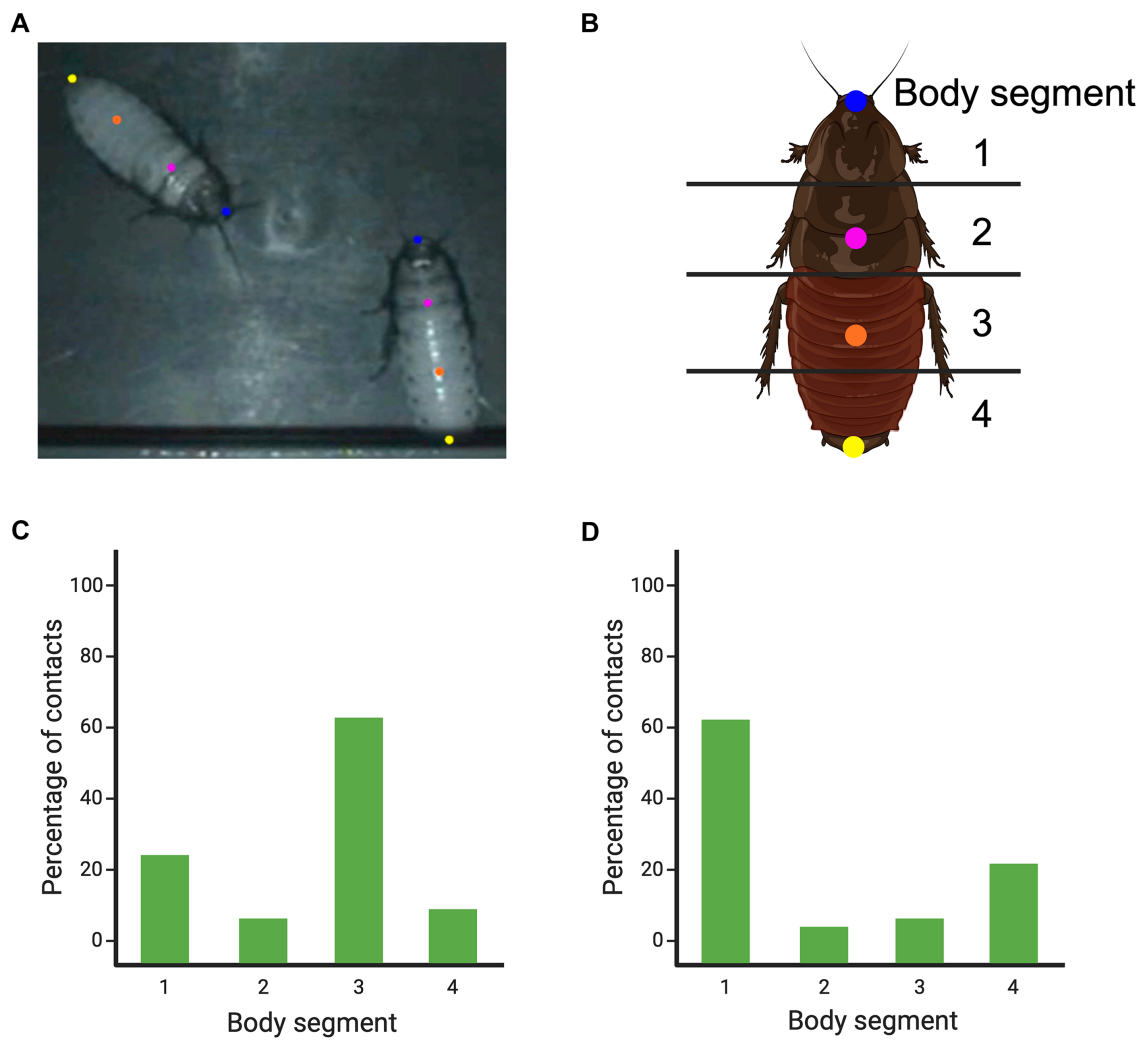
The body points that were tracked using DeepLabCut in pairs of Madagascar hissing cockroaches and the inter-animal orientations that were most frequently observed. Panel **(A)** shows an extracted and labeled frame from DeepLabCut, depicting two cockroaches and the body locations that were labeled. Panel **(B)** shows how the body was divided into four segments to score where the head and abdomen tip contacts were made [as devised by Bell (2014) and Pellis and Bell (2020)]. Panel **(C)** shows the percentage of head-to-body contacts that were made based on body segments. Panel **(D)** shows the percentage of tip of abdomen-to-body contacts that were made based on the body segments. Created, in part, with BioRender.com.

Using DeepLabCut (version 2.1.9) (Mathis et al., 2018; Nath et al., 2019), we labeled 200 frames from five videos (40 frames/video). Following previously identified body regions of interest (Bell, 2014; Pellis and Bell, 2020), we marked four body locations on the dorsum of each cockroach (Figures 10A,B). Individuals were identified, and labeled accordingly, by either the presence or absence of a dot of paint. Once the network was trained, we analyzed the videos. Using the X and Y coordinates, we determined where head-to-body contacts were made, relative to the marked body locations. We found a small modal rise for head-to-head contact and a large modal rise for the head-to-lower abdomen flank (Figure 10C). These results are consistent with those derived from manual scoring (Bell, 2014; Pellis and Bell, 2020).

Our results demonstrate that, once the initial patterns and configurations are understood through EWMN, DeepLabCut can be used to confirm or disconfirm the identified patterns. The added benefit is that once a neural network is sufficiently trained, hundreds of behavioral encounters/events can be processed by DeepLabCut or similar AI tracking software. Once processed, the pose estimations can then be used to confirm whether the predicted patterns and configurations derived from manual scoring hold true when hundreds of events (or more) are analyzed. However, an important step in this process is having first viewed and notated the behavior manually. Without knowing the basic pattern of behavior, you would not know which oppositions or joints are maintained and so which points of the body to track and which poses to score. We suggest that, for certain behavioral patterns, a combination of qualitative manual scoring and AI tracking may provide deeper insights into social behaviors than simply relying on AI methods alone. In turn, using AI methods can minimize the reliance on labor-and time-intensive manual scoring methods.

### Finding hidden patterns

Given the labor-intensive nature of using EWMN to track body parts and inter-animal configurations, what is notated is typically limited to what, at the outset, appears to be most likely to be important. For example, if the weapon system is situated at the front of the animal, then, in a combat setting, it would seem logical to track that weapon system, or the body part that is carrying that system, such as the head in animals using horns, antlers or teeth (Geist, 1966; Pellis, 1997), or the thoracic area behind the head carrying the horny protuberances used to grapple in fights by Madagascar hissing cockroaches (Fraser and Nelson, 1984). Therefore, when notating the combat sequences of the cockroaches, we focused on tracking the heads of the interactants and how they were oriented toward their opponent’s body (Bell, 2014; Pellis and Bell, 2020). In using DeepLabCut, however, we tracked the entire length of the animals’ bodies (four locations, Figures 10A,B), and, by trawling through the data on body configuration coordinates, we noted instances when the tip of the abdomen of one animal moved toward the body of the other animal. That is, we were directed to observing an opposition that we had missed using EWMN. This revelation had both methodological and biological implications.

Methodologically, what this did was provide an illustration of how EWMN and machine learning can be used iteratively. Based on EWMN, what is to be measured and tracked is determined, then the machine learning can not only test the patterns derived from EWMN but may reveal new patterns that need to be further investigated by EWMN and so on. Biologically, the iteration process led to a new interpretation of so-called cockroach abdominal flicks and pushes (Clark and Moore, 1994). Once one of the cockroaches gains a head-to-flank opposition and begins to lever its opponent’s abdomen upwards, the opponent resists by rotating that side of its body toward the ground (Pellis and Bell, 2020). However, as the attacker keeps maneuvering to overturn its opponent, the opponent reaches over and uses the tip of its abdomen to insert beneath the attacker’s body, and if successful, can flip the attacker over. Using the data derived from DeepLabCut to quantify the attacker’s body locations contacted by the tip of the defender’s abdomen, it was found that it was the head that was most frequently contacted and, to a lesser degree, the lower abdomen (Figure 10D). Closer, manual inspection of these contacts revealed that abdomen-to-head contact most often pushed the attacker away and abdomen-to-abdomen contact most often flipped the attacker over. These new insights call for a renewed, deeper analysis of combat in the cockroaches, but for present purposes, these findings highlight that, rather than scoring abdominal flicks/pushes and head butts as isolated, abstracted actions during fighting, considering them in a dynamic context of oppositions, the organization of the interactions come into sharper focus showing how those behavior patterns are used for attack and defense. Head butting can be seen as an offensive action when directed at the opponent’s flank, but as a defensive action when directed at the head, and abdominal flicks/pushes can be seen as retaliatory actions to reverse the advantage gained by the opponent.

## Conclusion

Interactions between animals are dynamic affairs that require methodologies that can track the actions of both animals over time. However, scoring predefined behavior patterns and then quantifying them to analyze sequential and temporal patterns may be insufficient to be able to contextualize those actions from the animals’ perspectives. Rather, the oppositions and joints that form the focal points around which interactions are organized (Golani, 1976) need to be identified and tracked as they change or remain fixed. It is only then that the animals’ uses of the behavior patterns identified by observers can be interpreted. Methods for such tracking, such as the EWMN, can be very labor intensive and so prohibitive for processing large quantities of videotaped material. However, as we have hopefully illustrated with the examples above, using a method like EWMN can be very helpful and so worth the effort. More encouragingly, modern methods of machine learning can augment the use of EWMN and not only reduce the amount of notation needed in any given study but can also be used to gain insights missed by EWMN, and so be a collaborative technology. Combining such qualitative and quantitative methods can provide both the detail that in-depth analysis of a small number of cases can achieve, with the benefits of testing relevance that can only be achieved by analyzing large data sets. Rather than replacing the innovative insights of Golani back in 1976, modern methods give new life to those insights.
